# Inflammation, Gut Microbiota, and Metabolomic Shifts in Colorectal Cancer: Insights from Human and Mouse Models

**DOI:** 10.3390/ijms252011189

**Published:** 2024-10-17

**Authors:** Chengcong Yang, Wusigale, Lijun You, Xiang Li, Lai-Yu Kwok, Yongfu Chen

**Affiliations:** 1 Key Laboratory of Dairy Biotechnology and Engineering, Ministry of Education, Inner Mongolia Agricultural University, Hohhot 010018, China; 18827513058@163.com (C.Y.); wusigale_imau@163.com (W.); youlijun1994@163.com (L.Y.); lxclaire0630@163.com (X.L.); kwok_ly@yahoo.com (L.-Y.K.); 2 Key Laboratory of Dairy Products Processing, Ministry of Agriculture and Rural Affairs, Hohhot 010018, China; 3 Inner Mongolia Key Laboratory of Dairy Biotechnology and Engineering, Inner Mongolia Agricultural University, Hohhot 010018, China

**Keywords:** tumor development, stool metagenome analysis, microbial dysbiosis, biomarkers, azoxymethane, dextran sulfate sodium

## Abstract

Colorectal cancer (CRC) arises from aberrant mutations in colorectal cells, frequently linked to chronic inflammation. This study integrated human gut metagenome analysis with an azoxymethane and dextran sulfate sodium-induced CRC mouse model to investigate the dynamics of inflammation, gut microbiota, and metabolomic profiles throughout tumorigenesis. The analysis of stool metagenome data from 30 healthy individuals and 40 CRC patients disclosed a significant escalation in both gut microbiota diversity and abundance in CRC patients compared to healthy individuals (*p* < 0.05). Marked structural disparities were identified between the gut microbiota of healthy individuals and those with CRC (*p* < 0.05), characterized by elevated levels of clostridia and diminished bifidobacteria in CRC patients (*p* < 0.05). In the mouse model, CRC mice exhibited distinct gut microbiota structures and metabolite signatures at early and advanced tumor stages, with subtle variations noted during the intermediate phase. Additionally, inflammatory marker levels increased progressively during tumor development in CRC mice, in contrast to their stable levels in healthy counterparts. These findings suggest that persistent inflammation might precipitate gut dysbiosis and altered microbial metabolism. Collectively, this study provides insights into the interplay between inflammation, gut microbiota, and metabolite changes during CRC progression, offering potential biomarkers for diagnosis. While further validation with larger cohorts is warranted, the data obtained support the development of CRC prevention and diagnosis strategies.

## 1. Introduction

Colorectal cancer (CRC) arises from the mucosa of the colon or rectum, primarily affecting the glandular cells within the colon [1]. It is currently the third most prevalent and second most lethal cancer worldwide [2]. Projections indicate that CRC will become the fastest-growing cancer globally in the coming decades, with an estimated 4.7 million cases expected by 2070 [3]. Early detection of CRC is challenging, as most cases are diagnosed during the advanced stages of tumor metastasis, which increases the likelihood of distant recurrence and decreases patient survival rates. Conversely, early detection through methods such as surgery can significantly improve the cure rate [4]. Therefore, understanding the etiology and progression of CRC is crucial. The pathogenesis of CRC involves multiple factors, including genetics [5], environment, and the microbiome [6]. Additionally, mutations in key tumor-suppressor genes and oncogenes (such as *KRAS* and *TP53*) within glandular and colon epithelial cells, along with chronic inflammation [7], contribute to the development of CRC.

In recent years, substantial evidence has highlighted the close connection between the gut microbiota and CRC [8]. The gut microbial community, residing within the intestinal tract, plays a vital role in host health and physiological functions [9]. Distinct gut microbiota signatures have been identified in CRC patients compared to healthy individuals, including alterations in specific bacterial genera, such as the reduction in the abundance of bifidobacteria [10]. It is hypothesized that the intestinal microbial community may be associated with the formation of precancerous lesions during the early stages of CRC. Gut dysbiosis, characterized by a decrease in beneficial bacterial species (such as butyrate-producing bacteria) and an increase in harmful bacterial populations (pro-inflammatory opportunistic pathogens), has been observed in CRC patients [11]. Certain gut microbes are implicated in chronic inflammatory processes that may further promote CRC progression [12]. Moreover, the specific composition of the gut microbiota may positively influence the treatment outcomes for CRC patients [13]. Therefore, investigating changes in the gut microbial community during CRC development can yield vital data to enhance CRC diagnosis and prognosis.

With the rapid advancement of sequencing technology and bioinformatics, metagenomic sequencing has become an indispensable tool for characterizing the gut microbial community [14]. It allows for a comprehensive analysis of microbial communities, offering insights into their diversity and functions and aiding in the identification of dysbiosis in disease contexts [15]. Furthermore, metagenomic technology facilitates the study of gut microbiota function, particularly in host metabolism, immune regulation, the synthesis of antibacterial metabolites, and other vital physiological roles of gut microbes [16]. This provides profound insights into gut homeostasis and its disruption in disease pathogenesis. Microbial community tracking via metagenomics also offers insights into how gut microbes respond to various treatment conditions and environmental factors. Analyzing these responses yields valuable information on disease progression and treatment strategies. Furthermore, metagenomic data analysis has the potential to identify biomarkers for the early diagnosis and risk assessment of gut-related diseases [17].

Such technology reveals the intricate interplay between the gut microbiota and human health, opening new avenues for understanding disease mechanisms and improving clinical outcomes. However, it is important to note that species abundance may not accurately reflect microbial function, partly due to ongoing adjustments in the nomenclature of many gut microbial species [18]. In fact, microbial gene function has been found to be more discriminative than species specificity in differentiating CRC from control samples [19]. Furthermore, the combined analysis of gut microbes and metabolites has gained significant attention as a diagnostic research focus. There is a current scarcity of research on the dynamics of CRC at the species, functional, and metabolite levels, underscoring the necessity for such studies in clinical settings.

This study hypothesizes that the host’s gut microbiota, along with its functions and metabolites, undergoes changes during the occurrence and progression of CRC, accompanied by aggravated systemic inflammation, which ultimately promotes tumor growth. To test our hypothesis, a CRC mouse model was established using azoxymethane (AOM) and dextran sulfate sodium (DSS). We analyzed the alterations in gut metagenomes and metabolomes at various time points during the onset and progression of CRC in these cancer-induced mice. The findings of this study provide compelling evidence of significant shifts in the host’s gut microbiota, functions, and metabolites throughout CRC development. Shedding light on their intricate relationships advances our understanding of CRC pathogenesis and may pave the way for improved diagnostic and therapeutic strategies.

## 2. Results

### 2.1. Gut Microbiota Composition in CRC Patients and Healthy Controls

**Figure 1:**
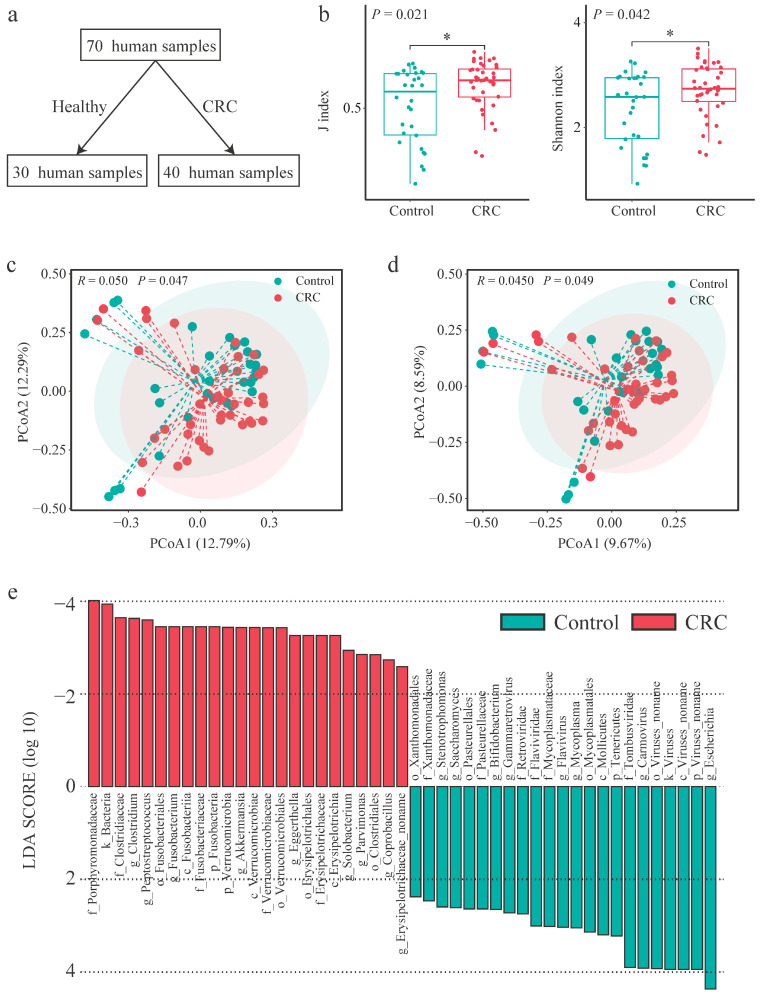
Gut metagenomics analysis of stool samples from colorectal cancer (CRC) patients and healthy controls (Control). (**a**) Sample size representation. (**b**) Alpha-diversity indexes: Shannon and J index. Statistical significance was determined using a *t*-test, with *p*-values indicated. (**c**,**d**) Principal coordinate analysis (Bray–Curtis and Jaccard distances) and (**e**) linear discriminant analysis (LDA) Effect Size (LEfSe) of fecal metagenomes from the two groups. Results of the Adonis test are shown in the principal coordinate analysis score plots. * *p* < 0.05.

Metagenomic data from 70 stool samples, including 30 from healthy controls and 40 from patients with CRC, were analyzed (Figure 1a). No significant disparities were observed between the two groups in terms of age, body mass index, and prevalence of underlying diseases (*p* > 0.05). The analysis disclosed notable differences in gut microbial diversity, with the Shannon and J indices revealing a notably higher microbial diversity in CRC patients compared to the control group (*p* < 0.05; Figure 1b). In addition, principal coordinate analysis (Bray–Curtis and Jaccard distances) showed a pronounced group-based clustering trend, although some overlap was evident on the score plots (*p* < 0.05, Adonis test; Figure 1c,d). Furthermore, the gut microbiota of CRC patients exhibited a significantly smaller intragroup variation compared to the healthy population (*p* < 0.05; Figure S1a,b). The majority of sequences in the gut samples were attributed to bacteria. However, bacteriophage sequences were also detected within the analyzed gut metagenome dataset (Figure S1c).

An examination of the gut microbial composition in both groups revealed that Bacteroidetes, Firmicutes, Proteobacteria, and Actinobacteria were the predominant bacterial phyla (Figure S1c), collectively constituting over 95% of the total microbial population. *Faecalibacterium prausnitzii*, *Prevotella copri*, and *Bacteroides stercoris* were identified as the most prevalent bacterial species (Figure S1d). The genera *Stenotrophomonas*, *Saccharomyces*, and *Bifidobacterium* were found to be more abundant in healthy individuals compared to CRC patients (Figure 1e). Conversely, the genera of *Clostridium*, *Peptostreptococcus*, and *Fusobacterium* were more prevalent in CRC patients compared to healthy individuals (Figure 1e). These results highlight the substantial differences in the structure and composition of the gut microbiota between CRC patients and healthy individuals.

### 2.2. Differential Gut Microorganisms Between the Cancer Group (CA Group) and Non-Cancer Group (Non-CA Group)

**Figure 2:**
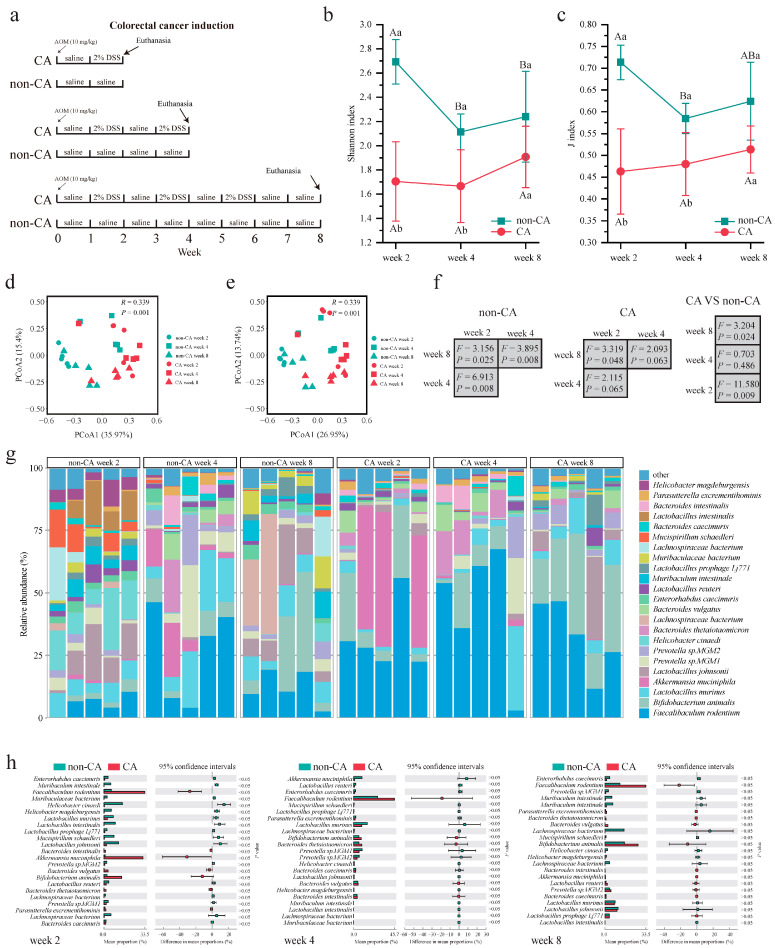
Metagenomics characterization of the gut microbiome in non-cancer control (non-CA) and cancer (CA) groups. (**a**) Schematic diagram showing the trial design, depicting the induction of colorectal cancer by administering azoxymethane (AOM) and dextran sulfate sodium (DSS). (**b**,**c**) Line charts representing the Shannon and J diversity indexes over time. Significant difference was evaluated using the ANOVA test. Within-group comparisons at different time points are denoted by capital letters, with the same letter indicating no significant difference (*p* > 0.05) and different letters indicating significant differences (*p* < 0.05). Between-group comparisons at the same time point are marked by lowercase letters, with the same letter indicating no significant difference (*p* > 0.05) and different letters indicating significant differences (*p* < 0.05). Error bars represent standard deviations. (**d**,**e**) Principal coordinate analysis based on Bray–Curtis and Jaccard distances. (**f**) *F*- and *p*-values generated by one-way PEMANOVA (Bray–Curtis distances). The left and middle panels show intragroup comparisons at different time points for the non-CA and CA groups, while the right panel displays intergroup comparisons at the same time point. (**g**) Relative abundance of dominant bacterial species in the mouse gut microbiota at various time points. (**h**) Abundance comparison of dominant bacterial species at weeks 2, 4, and 8, presented in horizontal bar charts at the left, middle, and right panels, respectively.

Numerous studies have established a close relationship between CRC development and alterations in the gut microbiota [20]. Utilizing an induced-cancer mouse model, this study investigated the dynamics of gut microbiota during the onset and progression of CRC (Figure 2a). The analysis revealed noteworthy trends in the gut microbiota of mice in the non-CA group (Figure 2b,c and Figure S2a). The Shannon, Simpson, J, and S indices exhibited a significant decrease from week 2 to week 4 (*p* < 0.05). However, no significant changes were observed from week 4 to week 8 (*p* > 0.05). In contrast, the gut microbiota of mice in the CA group did not exhibit significant changes from week 2 to week 4 (*p* > 0.05) but demonstrated a significant increase from week 4 to week 8 (*p* < 0.05). Comparatively, the Shannon and J indices of the gut microbiota in the non-CA group were significantly higher than those in the CA group at weeks 2 and 4 (*p* < 0.05), with no significant difference observed at week 8 (*p* < 0.05).

Furthermore, principal coordinate analysis (Bray–Curtis and Jaccard distances) revealed significant differences in the gut microbiota structure between mice in the non-CA and CA groups (*p* < 0.05; Figure 2d,e). As the experiment progressed, noticeable changes in the gut microbiota structure were observed in both groups (Figure 2f and Figure S2b). Notably, the gut microbiota structure of mice in the non-CA and CA groups significantly differed at weeks 2 and 8 (*p* < 0.05), with no significant difference at week 4 (*p* < 0.05; Figure 2f and Figure S2b).

Additional analysis of the gut microbiota composition in these mice indicated that the predominant bacterial phyla were Firmicutes, Bacteroidetes, Actinobacteria, and Verrucomicrobia (Figure S2c). The dominant bacterial species included *Faecalibaculum rodentium*, *Bifidobacterium animalis*, *Lactobacillus murinus*, *Akkermansia muciniphila*, and *Lactobacillus johnsonii* (Figure 2g). Key differential microbial species between the two groups were identified at three time points. At week 2, *Enterorhabdus caecimuris*, *Muribaculum intestinale*, *Muribaculaceae bacterium*, *Helicobacter cinaedi*, *Helicobacter magdeburgensis*, and *Lactobacillus murinus* were significantly more abundant in the non-CA group, while the relative abundance of *Faecalibaculum rodentium* was significantly lower (*p* < 0.05; Figure 2h). At week 4, no significant difference in dominant bacterial species was observed between the non-CA and CA groups (*p* < 0.05). By week 8, the relative abundances of intestinal *Enterorhabdus caecimuris* and *Prevotella* sp. *MGM1* were significantly higher in the non-CA group compared to the CA group (*p* < 0.05), with an opposite trend observed for *Faecalibaculum rodentium* (*p* < 0.05).

### 2.3. Variations in Gut Microbial Metabolic Pathways Between the CA and Non-CA Groups

We then comparatively analyzed the differences in gut microbial metabolic pathways between the CA and non-CA groups to reveal the association between colon cancer formation and the metabolic potential of the gut microbiome.

**Figure 3:**
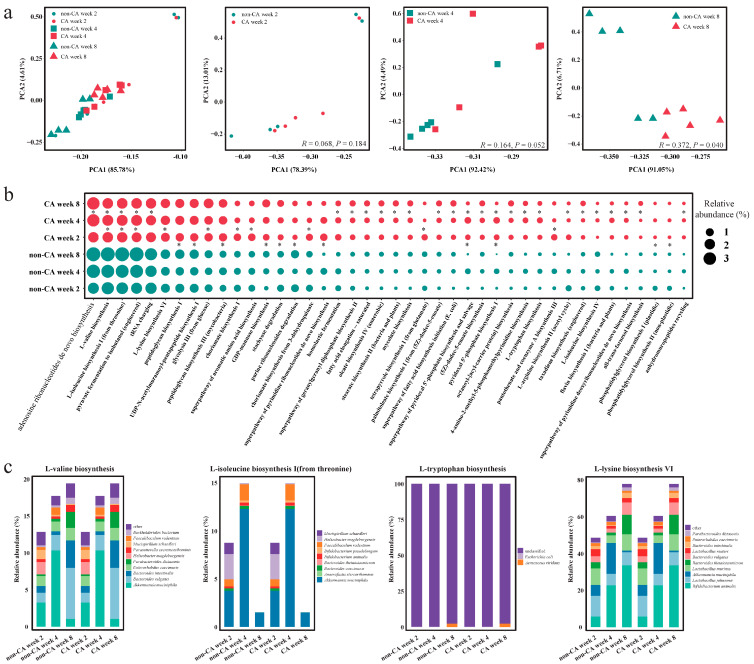
Differential gut microbial functional metagenomics analysis of the non-cancer (non-CA) and cancer (CA) groups. (**a**) Principal component analysis based on annotated gut microbial functional metagenomes. The left plot shows results from both groups at all three time points. The other plots depict intergroup comparisons at specific time points. (**b**) Dot plot showing differential metabolic pathways. The dot size represents relative abundance (%). Significant difference was determined by a *t*-test, with * *p* < 0.05 considered significant. (**c**) Distribution of genes encoding specific pathways across major species.

Principal component analysis revealed distinct group-based clustering patterns in the score plot (Figure 3a), with samples from the non-CA group primarily located in the lower left corner and those from the CA group mainly in the middle. When comparing the gut microbial metabolic pathways between the two groups at different stages, minor differences were observed at weeks 2 and 4, which were not statistically significant (*p* > 0.05). However, at week 8, a clear distinction was evident in the distribution of non-CA and CA groups on the left and right sides, respectively, indicating a divergence in their functional metagenome (*p* < 0.05). These results suggest that, as colon cancer progresses, the divergence in gut microbial pathways between the CA and non-CA groups intensifies gradually.

Further analysis identified key differential metabolic pathways at various time points (Figure 3b and Figure S3), reflecting the changes in gut microbial metagenomic potential during different stages of cancer progression. Consistent with the findings of the principal component analysis, the number of differential gut microbial metabolic pathways between the CA and non-CA groups increased with the advancement of colon cancer. These results highlight the gradual and dynamic nature of metabolic alterations during cancer development. At week 2, significant differences were observed in the pathways of peptidoglycan biosynthesis I, UDP-N-acetylmuramoyl-pentapeptide biosynthesis I, and peptidoglycan biosynthesis III (mycobacteria) between the two groups (*p* < 0.05). At week 4, significant differences were found in the pathways of pantothenate and coenzyme A biosynthesis III, glycolysis III (from glucose), and L-lysine biosynthesis VI between the two groups (*p* < 0.05). At week 8, significant differences were found in the pathways of L-isoleucine biosynthesis I (from threonine), L-tryptophan biosynthesis, and L-valine biosynthesis between the two groups (*p* < 0.05).

Additionally, we conducted a detailed analysis of some of the differential metabolic pathways. The results revealed that the pathways of L-valine biosynthesis, L-isoleucine biosynthesis I (from threonine), L-tryptophan biosynthesis, and L-lysine biosynthesis VI were associated with various bacterial species (Figure 3c).

### 2.4. Serum Levels of Cytokines, Lipopolysaccharide (LPS), and D-Lactic Acid (D-LA) in the CA and Non-CA Groups

**Figure 4:**
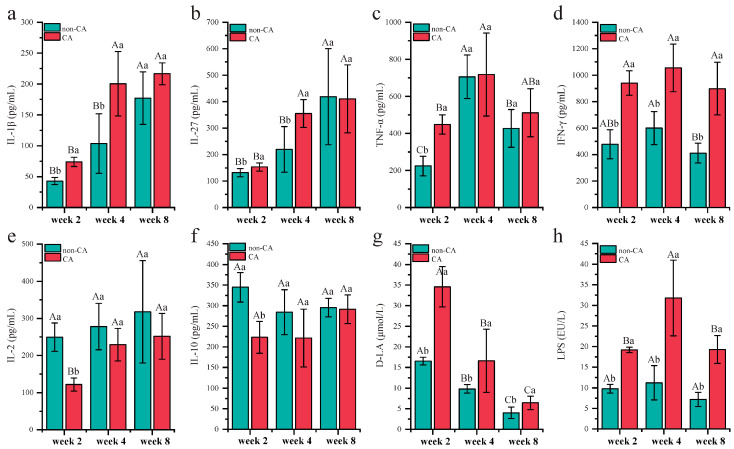
Serum levels of cytokines and biochemical indicators. (**a**) Interleukin (IL)-1β, (**b**) IL-27, (**c**) tumor necrosis factor-alpha (TNF-α), (**d**) interferon-gamma (IFN-γ), (**e**) IL-2, (**f**) IL-10, (**g**) D-lactic acid (D-LA), and (**h**) lipopolysaccharide (LPS). Non-CA and CA represent the non-cancer control and cancer groups, respectively. EU denotes endotoxin units. Significant difference was evaluated using the ANOVA test. Within-group comparisons at different time points are denoted by capital letters, with the same letter indicating no significant difference (*p* > 0.05) and different letters indicating significant differences (*p* < 0.05). Between-group comparisons at the same time point are marked by lowercase letters, with the same letter indicating no significant difference (*p* > 0.05) and different letters indicating significant differences (*p* < 0.05). Error bars represent standard deviations.

The onset and progression of CRC are intricately linked to the inflammation status of the host, with chronic inflammation identified as a pivotal contributor to CRC development [21]. Our results revealed a significant escalation in serum levels of interleukin (IL)-1β, IL-27, and tumor necrosis factor-alpha (TNF-α) in the CA group from week 2 to week 4 (*p* < 0.05; Figure 4a–c), with no significant variations detected at week 8 (*p* > 0.05). In addition, the levels of interferon-gamma (IFN-γ) were significantly elevated in the CA group at all assessed time points—weeks 2, 4, and 8 (Figure 4d; *p* < 0.05). Conversely, the levels of anti-inflammatory cytokines, namely, IL-2 and IL-10, were significantly reduced in the CA group relative to the non-CA group (*p* < 0.05; Figure 4e,f). These observations suggest a pronounced systemic inflammatory response characterized by elevated proinflammatory cytokines and diminished anti-inflammatory cytokines in the CA group.

To assess intestinal permeability, we evaluated the serum levels of D-LA and LPS. A notable decrease in D-LA levels was observed in both groups as the experiment progressed (*p* < 0.05; Figure 4g). However, the CA group consistently exhibited higher levels of both D-LA and LPS compared to the non-CA group throughout the study (*p* < 0.05; Figure 4h), indicating increased gut permeability in the CA group.

Principal component analysis of the serum factors revealed distinct clustering patterns between the CA and non-CA groups, highlighting differences in inflammatory profiles and gut permeability (Figure S4a). A detailed examination of the loadings plot identified key serum factors that significantly differentiated the two groups, including IL-10, IFN-γ, TNF-α, LPS, and D-LA (Figure S4b).

### 2.5. Differential Gut Metabolites Between the CA and Non-CA Groups

**Figure 5:**
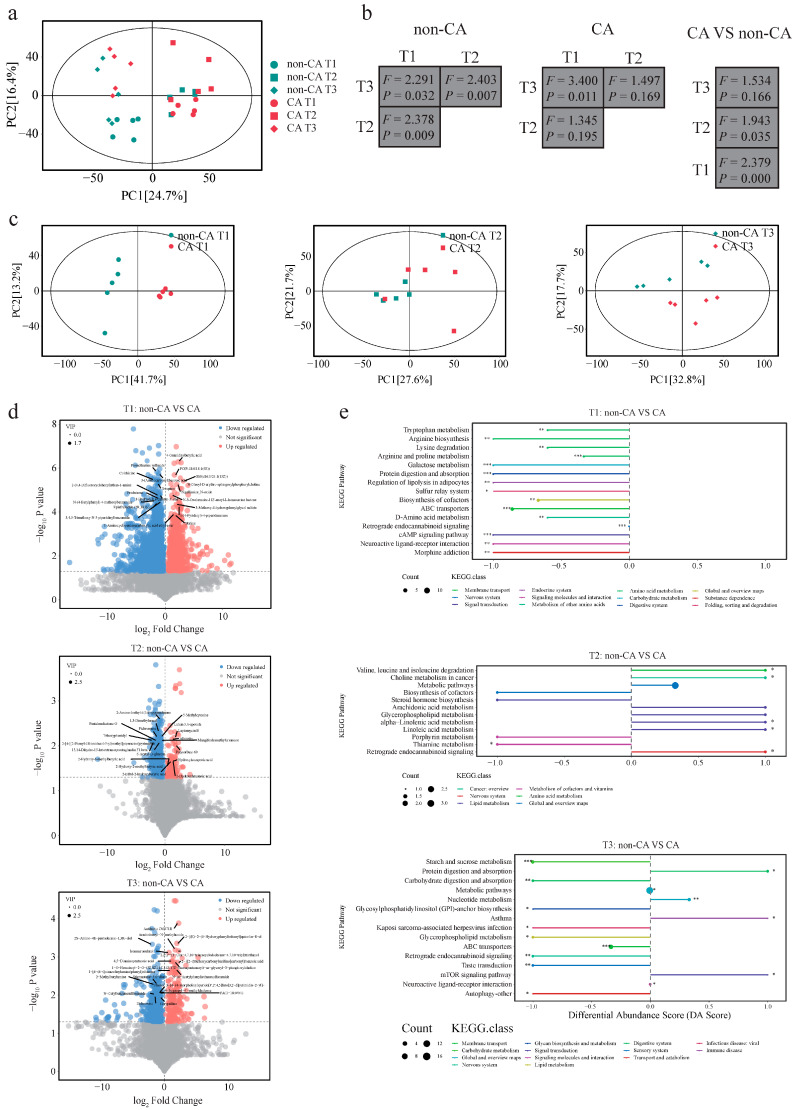
Analysis of the fecal metabolome of the non-cancer (non-CA) and cancer (CA) groups. (**a**,**c**) Principal component analysis based on the fecal metabolome. (**b**) One-way PERMANOVA analysis. (**d**) Volcano plots illustrating significantly increased, decreased, and non-significantly changed metabolites. (**e**) Pathway enrichment analysis of identified differential metabolites. A positive differential abundance (DA) score represents enrichment in the non-CA group, while a negative DA score represents enrichment in the CA group. The dot size represents the metabolite count in the specific pathway. * *p* > 0.05, ** *p* > 0.05, and *** *p* > 0.05.

Our study uncovered significant differences in the gut metabolome between mice in the non-CA and CA groups (Figure 5a–c and Figure S5a–c). To deepen our understanding of the metabolic shifts occurring in the gut during the development of CRC, a thorough examination of differential metabolites was conducted. A total of 3301, 484, and 805 differential metabolites were identified between the non-CA and CA groups at weeks 2, 4, and 8, respectively (Figure 5d, *p* < 0.05). The Venn diagram in Figure S5d illustrates the number of differential metabolites present in the CA and non-CA groups.

Subsequently, an enrichment analysis was performed on the identified differential metabolites at the pathway level (Figure 5e). At week 2, the CA group showed significantly reduced levels in pathways associated with amino acid metabolism and energy metabolism, including tryptophan metabolism, arginine biosynthesis, and arginine and proline metabolism (*p* < 0.05). By week 4, the CA group exhibited significantly elevated levels in pathways such as valine, leucine, and isoleucine degradation; choline metabolism in cancer; and linoleic acid metabolism (*p* < 0.05). At week 8, the CA group demonstrated significantly diminished levels in pathways of starch and sucrose metabolism, carbohydrate digestion and absorption, retrograde endocannabinoid signaling, and autophagy—other (*p* < 0.05). Conversely, pathways of nucleotide metabolism, asthma, and mTOR signaling were significantly more abundant in the CA group (*p* < 0.05).

### 2.6. High Specificity of Gut Microbial Species, Pathways, Metabolites, and Serum Indicators in CRC Prediction

**Figure 6:**
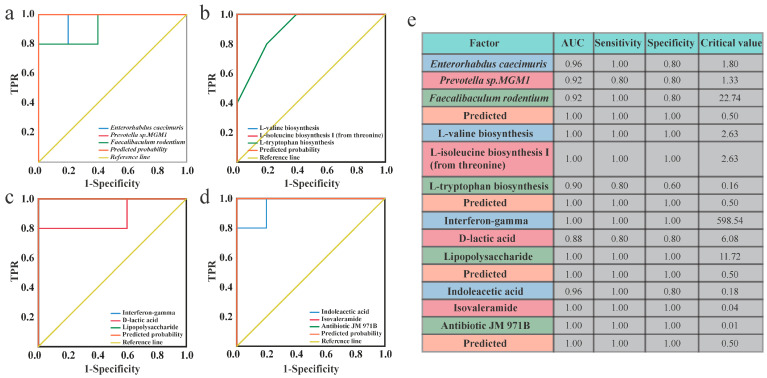
Receiver operating characteristic (ROC) curve analysis. (**a**–**d**) ROC curves were constructed for biomarkers including (**a**) species, (**b**) pathways, (**c**) inflammatory factors, and (**d**) metabolites. (**e**) The area under the curve (AUC), sensitivity, specificity, and critical value for each factor are shown. TPR stands for true positive rate.

To identify potential biomarkers for CRC, we constructed receiver operating characteristic curves incorporating data on differential gut microbial species, metabolic pathways, metabolites, and serum indicators (Figure 6a–d). We calculated the area under the curve, sensitivity, specificity, and critical value to assess the predictive accuracy of each factor and its capacity to distinguish CRC (Figure 6e). Our results revealed several factors with robust discriminatory power for CRC, characterized by a high area under the curve (ranging from 88 to 100%), sensitivity (80 to 100%), and specificity (60 to 100%). These factors included differential gut microbial species such as *Enterorhabdus caecimuris*, *Prevotella* sp. *MGM1*, and *Faecalibaculum rodentium*; metagenomic pathways like L-valine biosynthesis, L-isoleucine biosynthesis I (from threonine), and L-tryptophan biosynthesis; serum factors including IFN-γ, D-LA, and LPS; and gut metabolites such as indoleacetic acid, isovaleramide, and antibiotic JM971B. Collectively, these findings underscore the significant association between CRC development and specific gut microbial species, metagenomic pathways, serum factors, and gut metabolites, emphasizing their potential as precise and pertinent biomarkers for CRC.

## 3. Discussion

The gut microbiota and its metabolites play a critical role in maintaining host health, including the regulation of immunity, inflammatory responses, metabolism, and gut barrier function [22,23]. Gut dysbiosis has been associated with an increased susceptibility to tumors and can inform clinical treatment strategies [24]. Consequently, assessing the equilibrium of gut microbial populations and metabolic levels may yield valuable insights for cancer prevention [25]. In this study, we integrated clinical data analysis with an animal study to investigate the characteristics of the gut microbiota in CRC. We utilized an induced colon cancer mouse model to monitor changes in gut metagenomics and metabolomics in relation to cancer progression.

Our initial analysis compared the gut microbial metagenome between CRC patients and healthy individuals, corroborating our findings with the CRC mouse model. Consistently, we observed significant alterations in microbial diversity, community structure, and the prevalence of key bacterial species associated with CRC [18]. However, it is noteworthy that CRC patients exhibited higher microbial diversity than in healthy individuals, contrasting with the mouse model, where no significant differences in microbial diversity were observed post-tumor formation. These results align with previous reports [10,26]. The discrepancy between human gut metagenome data and the animal model may be attributed to disparate host factors, environmental conditions, and lifestyle choices [27]. Nonetheless, these findings underscore the potential of monitoring gut microbiota alterations as a viable strategy for early CRC diagnosis and intervention.

Host inflammatory levels are intricately connected to CRC development. Our study found a higher relative abundance of intestinal bifidobacteria in healthy individuals compared to CRC patients. Some bifidobacteria have been shown to notably diminish systemic inflammation and enhance gut barrier integrity, and alterations in gut bifidobacteria levels may have direct or indirect effects on tumorigenesis [28]. Additionally, we observed increased levels of fecal *Clostridium* and *Fusobacterium* in CRC patients. Specific *Clostridium* species, such as *Clostridium difficile*, are prevalent gut pathogens implicated in the progression of rectal cancer and can trigger severe gut infections [29]. *Fusobacterium nucleatum* can facilitate CRC development through multiple mechanisms, including the promotion of tumor cell proliferation, suppression of immune responses, and induction of inflammation. Elevated levels of *Fusobacterium nucleatum* have been correlated with poor prognosis and tumor recurrence in CRC patients [30]. Beyond variations in bacterial composition, we also noted a higher prevalence of bacteriophage sequences in the gut microbiota of healthy subjects compared to CRC subjects. Bacteriophages are integral components of the gut microbiome [31], exerting a positive influence on stability and overall health. As viruses that target bacteria, bacteriophages may be instrumental in modulating the diversity and stability of intestinal bacterial communities [32]. It has been established that bacteriophages can influence host health by regulating bacterial populations and modifying bacterial metabolic pathways [33]. Therefore, alterations in the intestinal phageome composition and structure may disrupt the balance of the gut microbiome and undermine its overall ecological equilibrium. However, it is crucial to acknowledge that while these observations offer significant insights, further comprehensive research is necessary to clarify the mechanisms of phage–bacteria interactions within the gut microbiota and their potential role in CRC.

An intriguing revelation from this study is the dynamic nature of gut microbial diversity and composition across various stages of CRC development, shedding light on the complex interplay between the disease and the microbial ecosystem. The gut microbiota diversity and structure in cancer mice exhibited variations at different phases of cancer progression. The most pronounced differences were observed in the early stages, with diminishing variations in the intermediate stages and a further reduction in the advanced stages. This dynamic modulation and adaptation of the gut microbiome during CRC development may reflect the effects of cellular mutations, inflammation responses, and changes in the gut microenvironment, such as mucosal damage, resulting in disturbances and potentially a reconfiguration of the gut microbiome [34]. During the intermediate stages of colon cancer formation, the gut microbiota accommodated to the microenvironment, minimizing differences in microbial composition. This adaptation underscores the responsive plasticity of the gut microbiota to the evolving conditions of colorectal cancer. However, as CRC advanced, the volume and mass of tumors escalated, inflicting further harm on the gut microenvironment and disrupting the gut microbial ecosystem. It is noteworthy that *Enterorhabdus caecimuris* exhibited a significant decrease in the early and late stages, accompanied by a significant increase in *Faecalibaculum rodentium*. *Enterorhabdus caecimuris* has been linked to processes such as gut inflammation, immune dysregulation, and CRC progression [35]. Differential pathway analysis indicated that fewer functional gut metagenome disparities existed between CRC and healthy mice in the early and middle stages of CRC development. Nevertheless, as CRC progressed to later stages, an increasing number of differential pathways were identified, hinting at a potential divergence between changes in microbial community structure and metabolic shifts. We speculate that this divergence may arise because different microbial species might contribute to the same metabolic pathway, suggesting that alterations in microbial community structure might not invariably align with corresponding changes in metabolic activity. This study highlights the complexity of the gut microbiota and its potential involvement in disease progression.

Our findings revealed a significant association between CRC development and heightened systemic inflammation, as evidenced by the notable increase in serum levels of proinflammatory cytokines, including IL-1β and TNF-α, alongside a reduction in the anti-inflammatory cytokine IL-10. Persistently elevated inflammation can induce mucosal damage, aberrant cell proliferation, and genetic mutations [36]. This inflammatory process activates and releases cells and mediators that can stimulate tumor cell growth, metastasis, and angiogenesis [37] while simultaneously impairing immune surveillance and facilitating tumor evasion [38]. Additionally, our data unveiled an interesting trend in normal mice, with age-related increases in cytokine levels such as IL-1β, IL-27, and IL-2. This observation may reflect a natural process of immunosenescence or chronic low-grade inflammation [39].

Gut metabolites have been recognized as pivotal in the etiology and progression of CRC through multiple pathways [39]. Our analysis of gut metabolites revealed trends that paralleled those of the bacterial microbiota during CRC development. The gut microbiota actively participates in diverse metabolic pathways, such as carbohydrate, amino acid, and short-chain fatty acid metabolism, leading to the production of diverse metabolites. Different compositions of the gut microbial community can influence its metabolism [23]. Our findings indicated a significant decrease in energy and amino acid metabolism levels within the intestinal milieu of mice during the early and late stages of CRC development. A previous study correlated the downturn in energy metabolism with damage to and dysfunction of the gut mucosal barrier, increasing its vulnerability to external stimuli and carcinogens [40]. Moreover, diminished energy metabolism can exacerbate chronic inflammation [41], a key driver in CRC development. Furthermore, the tryptophan metabolic pathway was notably enriched in the gut metagenome of the CA group. L-tryptophan, an essential amino acid, is engaged in metabolic pathways that influence tumorigenesis. Enzymes that metabolize tryptophan, such as tryptophanase and tryptophan hydroxylase, can influence tumor growth, metastasis, and angiogenesis. Tryptophan also modulates immune responses to tumors by regulating T-cell activity and immune cell function within the tumor microenvironment [42], potentially impacting tumor progression and treatment outcomes.

This study offers preliminary insights into the gut microbial characteristics of CRC patients compared to healthy individuals. It also highlights the dynamic shifts in gut microbiota structure, metabolites, and inflammation levels throughout CRC development using a mouse model. However, due to the limited sample size in this study, these patterns require further exploration. Future research with larger cohorts is warranted to validate and expand upon the current findings.

## 4. Materials and Methods

### 4.1. Bioinformatic Analysis of Human Fecal Microbial Metagenomes

This study analyzed metagenomic sequence data retrieved from a publicly accessible genome database comprising 70 human stool samples (30 from healthy individuals and 40 from CRC patients). We specifically selected data from early onset CRC patients to align with the focus of this study on the initial stages of CRC in 6-week-old male C57BL/6J mice. The 70 patients were screened based on stringent inclusion criteria, including a diagnosis of early onset CRC (age < 50 years), complete availability of gut microbiome data, and comprehensive clinical information.

The fecal metagenomic data analysis was conducted utilizing the HMP Unified Metabolic Analysis Network 3 (HUMAnN3) [43]. First, the metagenomic data underwent quality control using FastQC software (version 0.12.0), which involved the removal of host genome and low-quality sequences [44]. Then, MetaphlAn 3 (version 3.0) was used to calculate the relative abundance of various microbial groups within the metagenome dataset [45]. Bowtie 2 (ver 2.3) software [46] and the ChocoPhlAn (ver 3.0.1) [47] database were used to verify the nucleotide alignment of the pan-genome at the species level. Alpha diversity was calculated using R software (version 4.0.4; https://www.r-project.org/; accessed on 17 February 2024) and the vegan package [48]. Unpaired sequences were further aligned against the UniRef90 database using Diamond (ver 2.1.9) software and translated into putative protein sequences [49]. This approach facilitated a microbial functional metagenome analysis at the gene family and pathway levels.

### 4.2. Ethics Statement

The Ethics Committee of Inner Mongolia Agricultural University provided approval for all animal experimental procedures (approval No. NND2023094).

### 4.3. Mice and Diet

Thirty 5-week-old male C57BL/6J mice were purchased from Beijing Speford Laboratory Animal Technologies Co., Ltd. (Beijing, China). The mice were acclimated in the animal facility before the study started, with unrestricted access to water and a standard chow diet throughout this period. The animals were maintained in a specific pathogen-free environment with controlled conditions: a temperature of 21 °C, a 12 h light/dark cycle, and 50% humidity.

### 4.4. Trial Design

Following the acclimatization period, the 30 mice were randomly allocated into two groups: the non-CA group (*n* = 15) and the CA group (*n* = 15). The cancer model was induced by administering AOM and DSS. The CA group received an intraperitoneal injection of 0.2 mL AOM on day 0 (10 mg/kg per mouse; Sigma-Aldrich, Darmstadt, Germany), while the non-CA group was administered 0.2 mL of intraperitoneal normal saline. Subsequently, the CA group was administered 2% DSS (MP Biomedicals, Santa Ana, CA, USA) in drinking water during weeks 2, 4, and 6 [50]. At weeks 2, 4, and 8, five animals from each group were euthanized to collect serum, feces, and other relevant samples.

### 4.5. Confirmation of Colorectal Cancer Model

We analyzed tumor samples from the mouse colon. The intestinal tissue shows an adenoma characterized by tubular structures lined with dysplastic epithelium. The glandular structures are irregular and tightly packed, with epithelial cells featuring large, irregular, and highly basophilic nuclei. Occasional mitotic features are observed (indicated by black arrows). We also observed a small number of necrotic cell fragments within the glandular lumens (indicated by red arrows). Two areas exhibit mucosal erosion and necrosis of the intestinal glands in the lamina propria, leading to the replacement of these structures with proliferative connective tissue (indicated by green arrows). Additionally, metaplasia of the mucosal epithelium is observed, with columnar epithelium transforming into squamous epithelium in some regions (indicated by yellow arrows).

### 4.6. Analysis of Serum Levels of Cytokines, LPS, and D-LA

Serum levels of cytokines, including IL-10, IL-27, IL-2, IL-1β, TNF-α, and IFN-γ, as well as LPS and D-LA, were measured using enzyme-linked immunosorbent assay (ELISA) following the manufacturer’s protocols (Ruixin Biotechnology Co., Ltd., Quanzhou, China). The absorbance of the ELISA plates was measured at a wavelength of 450 nm using an ELISA plate reader (Ruixin Biotechnology Co., Ltd., Quanzhou, China).

### 4.7. Fecal DNA Extraction and Metagenomic Shotgun Sequencing

Metagenomic DNA was extracted from thawed fecal samples using the QIAamp DNA Stool Mini Kit (Qiagen, Hilden, Germany) following the manufacturer’s instructions. The purity and concentration of the extracted DNA were assessed using agarose gel electrophoresis and UV spectrophotometry. The NEBNext^®^ Ultra™ DNA Library Prep Kit for Illumina (New England Biolabs, Ipswich, MA, USA) was utilized to prepare a sequencing library from 1 μg of DNA, following the manufacturer’s instructions. Upon completion of library construction, preliminary quantification was conducted using Qubit 2.0, after which the library was diluted to 2 ng/μL. The insert size of the library was verified using the Agilent 2100 Bioanalyzer system (Agilent Technologies, Inc., Santa Clara, CA, USA). Once the insert size met the expected criteria, quantitative polymerase chain reaction was employed to estimate the effective concentration, ensuring a value greater than 3 nM. Qualified libraries were pooled for sequencing on an Illumina HiSeq X Ten platform to generate paired-end reads of approximate length of 2 × 150 bp [51].

### 4.8. Metabolomics Analysis

Animal tissue samples (24 mg) were combined with homogenizer beads and 500 μL of extraction solution (MeOH:ACN:H_2_O, 2:2:1 [*v*/*v*]) containing deuterated internal standards. The mixture was vortexed for 30 s and homogenized (35 Hz, 4 min) before undergoing sonication for 5 min in a 4 °C water bath. This process was repeated three times. The mixture was then vortexed for 30 s, sonicated for 10 min in a 4 °C water bath, and incubated for 1 h at −40 °C to precipitate proteins. The samples were then centrifuged at 12,000 rpm (RCF = 13,800× *g*, R = 8.6 cm) for 15 min at 4 °C. The supernatant was transferred to a sample vial for subsequent analysis. A quality control sample was prepared by pooling equal aliquots of the supernatant from all samples.

For the analysis of polar metabolites, LC-MS/MS analyses were conducted using a Vanquish UHPLC system (Thermo Fisher Scientific Inc., Waltham, MA, USA) coupled with an Orbitrap Exploris 120 mass spectrometer (Thermo Fisher Scientific Inc., Waltham, MA, USA) and a Waters ACQUITY UPLC BEH Amide column (2.1 mm × 50 mm, 1.7 μm; Water Corporation, Milford, MA, USA). The mobile phase consisted of 25 mmol/L of ammonium acetate and 25 mmol/L of ammonia hydroxide in water (pH = 9.75) (A) and acetonitrile (B). The auto-sampler temperature was maintained at 4 °C, and the injection volume was set to 2 μL. The Orbitrap Exploris 120 mass spectrometer was used because of its ability to acquire MS/MS spectra in information-dependent acquisition mode, which was controlled by the acquisition software, Xcalibur (Ver 4.0; Thermo Fisher Scientific Inc., Waltham, MA, USA). In this mode, the acquisition software continuously evaluated the full scan MS spectrum. The electrospray ionization source conditions were as follows: sheath gas flow rate, 50 Arb; Aux gas flow rate, 15 Arb; capillary temperature, 320 °C; full MS resolution, 60,000; MS/MS resolution, 15,000; collision energy, SNCE 20/30/40; spray voltage, 3.8 kV (positive ion mode) or −3.4 kV (negative ion mode), respectively.

The raw data were converted to the mzXML format using ProteoWizard and processed using an in-house program developed in R. The program was based on XCMS and utilized for peak detection, extraction, alignment, and integration. Metabolite identification was performed using the R package and the BiotreeDB (version 3.0) database [52].

### 4.9. Data Availability

The sequence dataset was deposited in the NCBI Sequence Read Archive (SRA) database (accession number: PRJNA1123426, https://www.ncbi.nlm.nih.gov/bioproject/1123426; accessed on 21 December 2023). Moreover, this study analyzed the metagenomic sequence data of 70 human stool samples, including 30 from healthy individuals and 40 from CRC patients. The data were retrieved from the National Center for Biotechnology Information Sequence Read Archive database with the accession number PRJNA763023 (https://www.ncbi.nlm.nih.gov/bioproject/763023; accessed on 21 December 2023) [10].

### 4.10. Statistical Analysis

Statistical differences in microbial abundance and alpha diversity between groups were evaluated using the Wilcoxon rank sum test. Statistical differences in inflammatory cytokine levels between groups were assessed using the analysis of variance (ANOVA) test. The *t*-test was used to determine statistical differences in metabolite levels between groups. The data were visualized using R software (Ver 4.0.4).

## 5. Conclusions

In conclusion, our study confirms the existence of CRC-associated gut dysbiosis through the analysis of human metagenomics data, emphasizing its significance in tumor progression. Significant alterations in systemic inflammation, gut permeability, and the gut metagenome and metabolome were observed in an AOM-DSS CRC mouse model, suggesting their potential involvement in the initiation and progression of CRC. Utilizing receiver operating characteristic curve analysis, we identified potential biomarkers that exhibit high specificity for the early detection of CRC. These findings offer valuable insights for the development of diagnostic markers, targeted therapies, and personalized treatment approaches to optimize CRC management. Further validation of these findings is warranted.

## Data Availability

Data is contained within the article and Supplementary Materials.

## Supplementary Materials

The following supporting information can be downloaded at: https://www.mdpi.com/article/10.3390/ijms252011189/s1.
