# Supplementary material for: Inflammation, Gut Microbiota, and Metabolomic Shifts in Colorectal Cancer: Insights from Human and Mouse Models
Source: Int J Mol Sci. 2024 Oct 17;25(20):11189. doi: 10.3390/ijms252011189 (PMC11508446; doi:10.3390/ijms252011189)
Supplement: Supplementary file 1 [file ijms-25-11189-s001.zip › Suppl. Figure captions.docx]

**Figure S1** Gut microbiome diversity in patients with colorectal cancer (CRC) and healthy controls (Control). (a, b) Intra- and intergroup Bray-Curtis and Jaccard distance analyses of the gut microbiome. (c, d) Relative abundances of dominant phyla and genera in the gut microbiota of the two groups.

**Figure S2** Gut microbiome diversity in patients with non-cancer control (non-CA) and cancer (CA) groups. (a) Line chart showing Simpson’s diversity and Shannon index. Significant difference was evaluated using the ANOVA test. Within-group comparisons at different time points are denoted by capital letters, with the same letter indicating no significant difference (*P* > 0.05) and different letters indicating significant differences (*P* < 0.05). Between-group comparisons at the same time point are marked by lowercase letters, with the same letter indicating no significant difference (*P* > 0.05) and different letters indicating significant differences (*P* < 0.05). Error bars represent standard deviations. (b) *F*- and *P*-values generated by one-way PEMANOVA (Jaccard distance). The results of intragroup comparison between different time points for the non-CA and CA groups (left and middle panel) and intergroup comparison at the same time point (right panel) are shown. (c) Relative abundance of dominant bacterial phyla in the gut microbiota of the two groups at different ime points.

**Figure S3** Dot plot showing differential metabolic pathways. The size of each dot represents the relative abundance (%). Significant difference was determined using a t-test, with statistical significance set at *P* < 0.05 (* *P* > 0.05, ** *P* > 0.05, and *** *P* > 0.05).

**Figure S4** Principal component analysis of serum cytokine and biochemical factor levels. (a) Score plot and (b) loadings plot. Non-CA and CA represent the non-cancer control group and the cancer group, respectively.

**Figure S5** Principal component analysis of the fecal metabolomes of patients with non-cancer control (non-CA) and cancer (CA) groups at different time points. Score plots of analysis of (a) both groups, (b) non-CA group, and (c) CA group. QC represents quality control samples. (d) Venn diagram of differential metabolites.
