# Supplementary figures and images for: Inflammation, Gut Microbiota, and Metabolomic Shifts in Colorectal Cancer: Insights from Human and Mouse Models
Source: Int J Mol Sci. 2024 Oct 17;25(20):11189. doi: 10.3390/ijms252011189 (PMC11508446; doi:10.3390/ijms252011189)

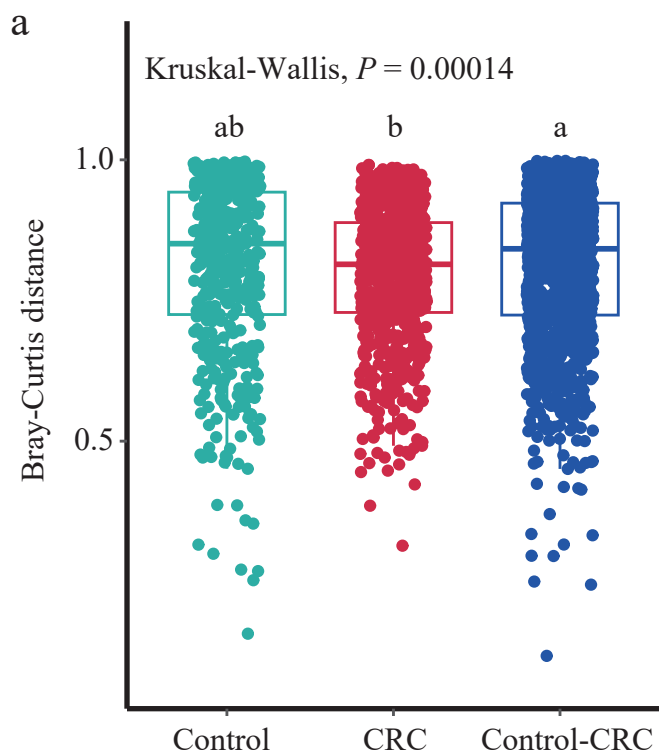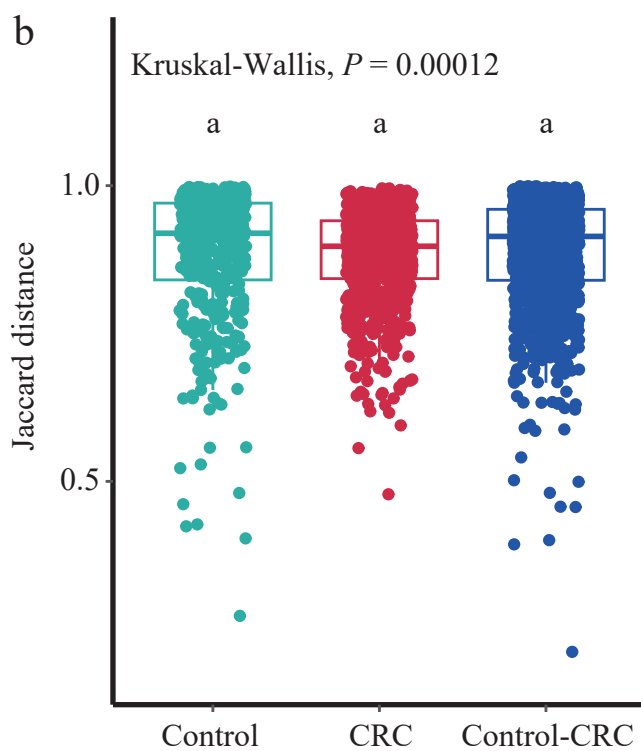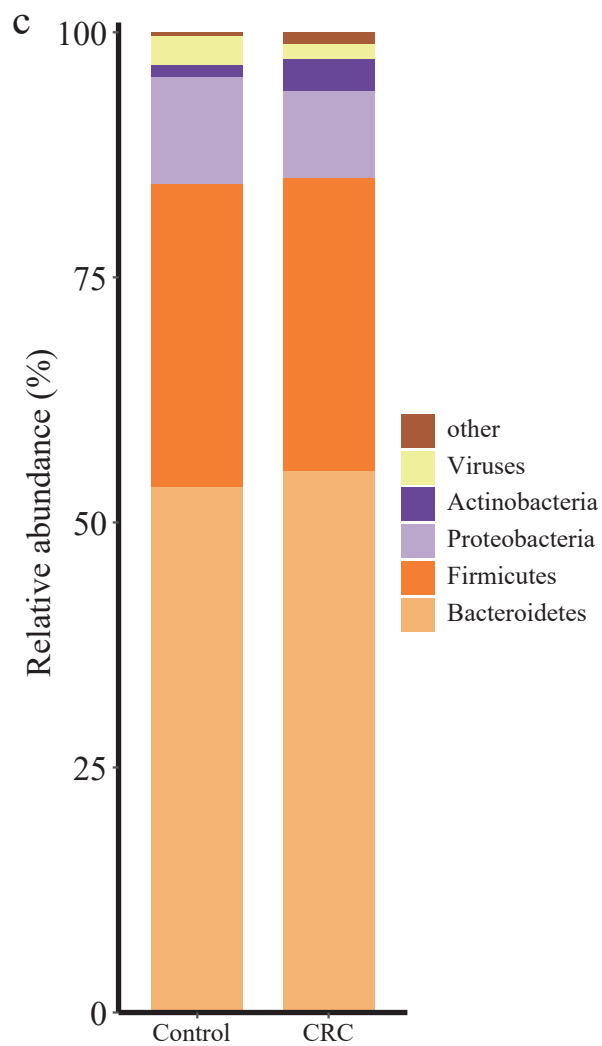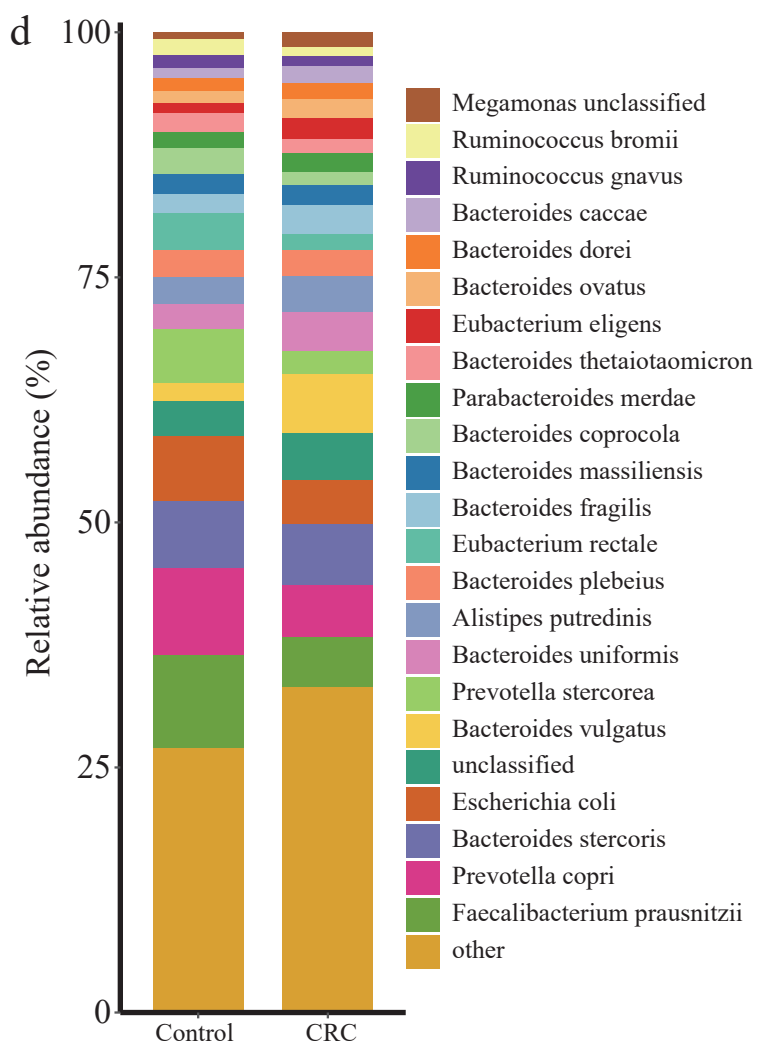

Supplement: Supplementary file 1 [file ijms-25-11189-s001.zip › Figure S1.pdf]

a

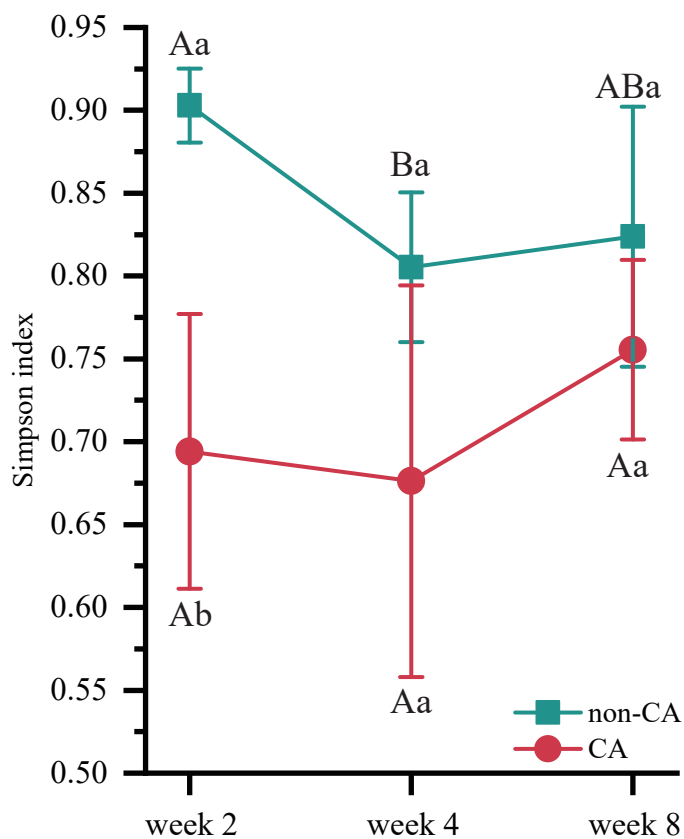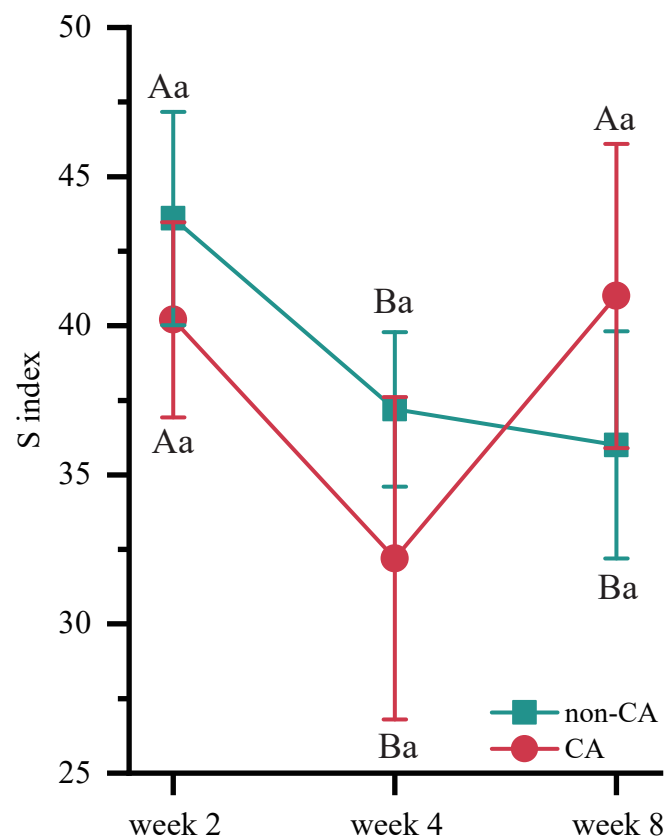

CA VS non-CA

b

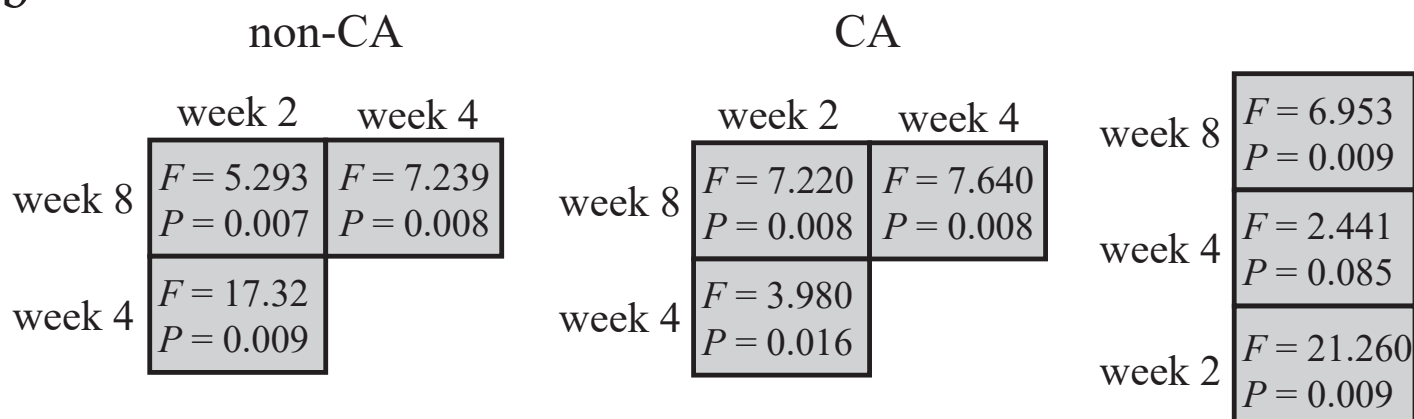

c

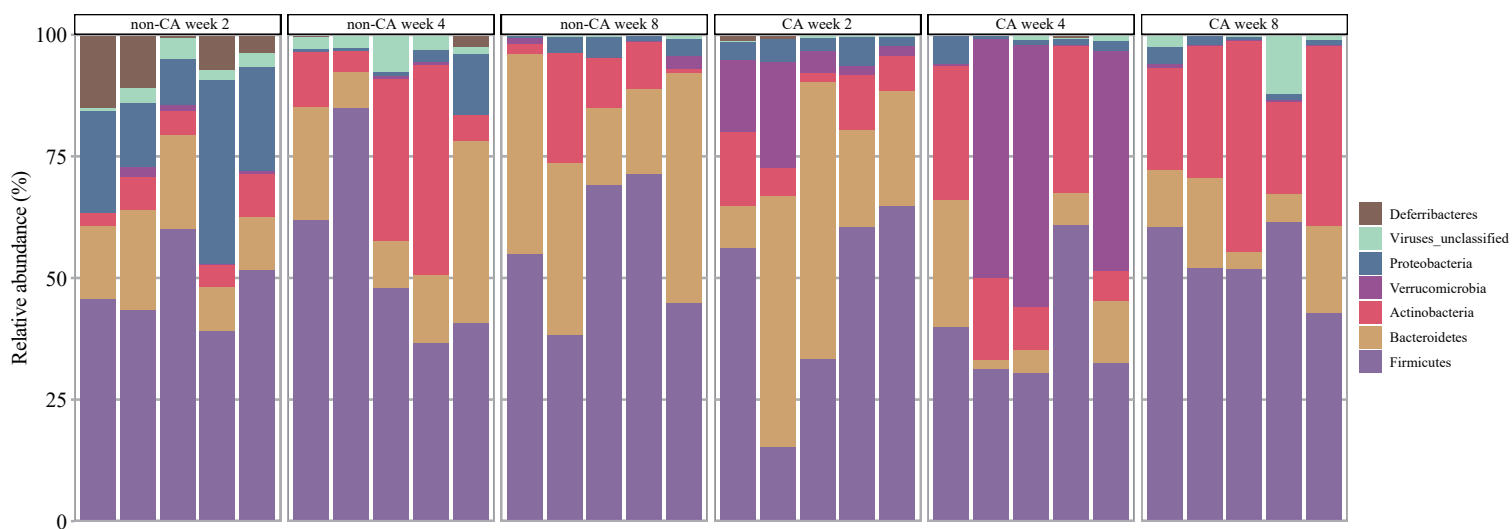

Supplement: Supplementary file 1 [file ijms-25-11189-s001.zip › Figure S2.pdf]

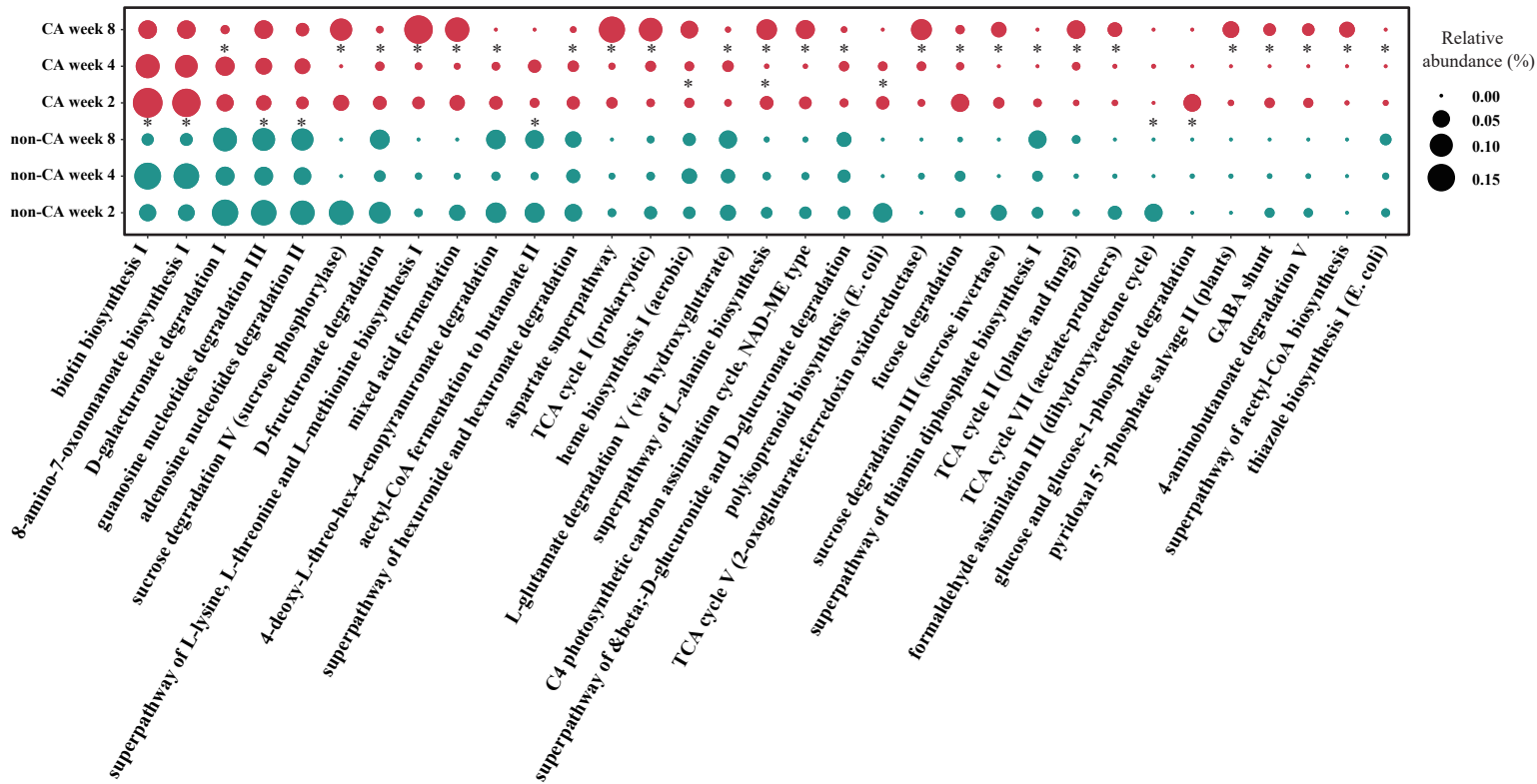

Supplement: Supplementary file 1 [file ijms-25-11189-s001.zip › Figure S3.pdf]

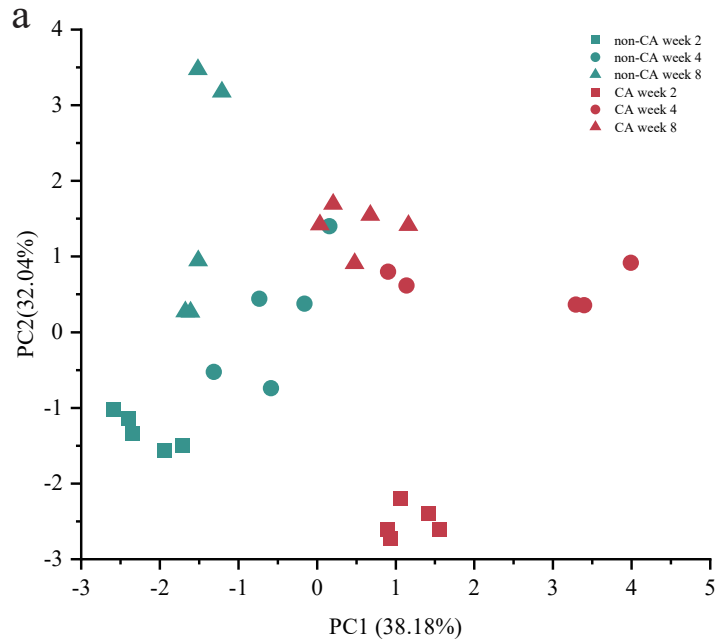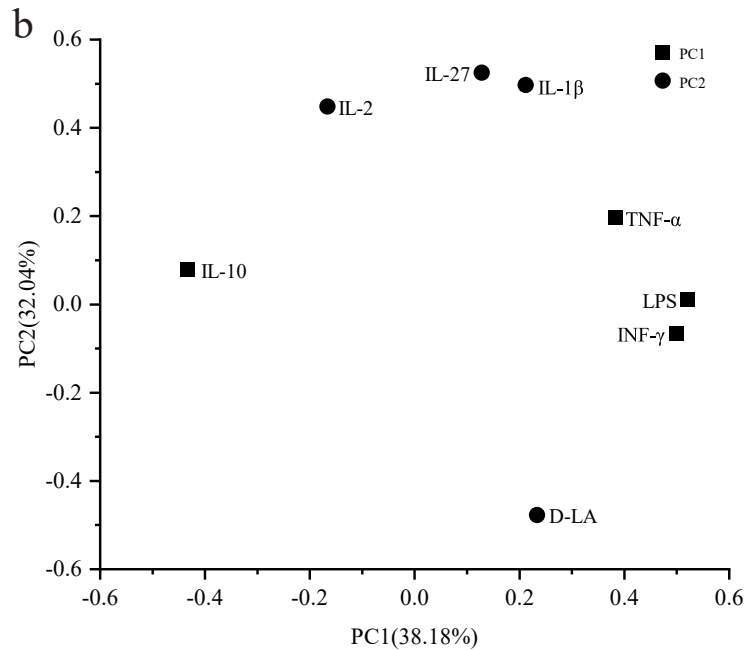

Supplement: Supplementary file 1 [file ijms-25-11189-s001.zip › Figure S4.pdf]

a

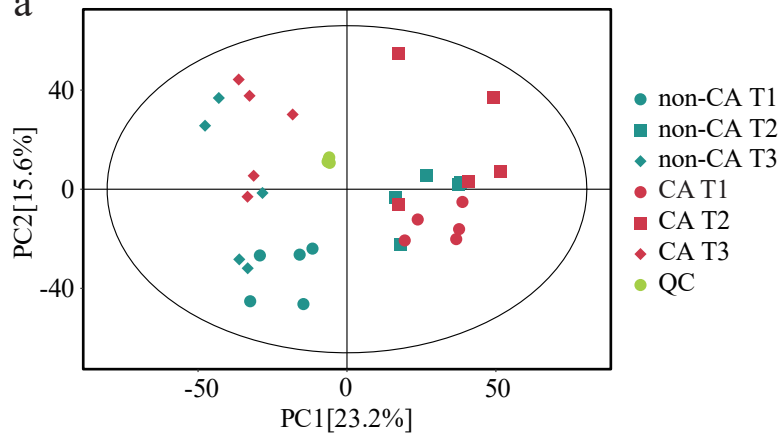

b

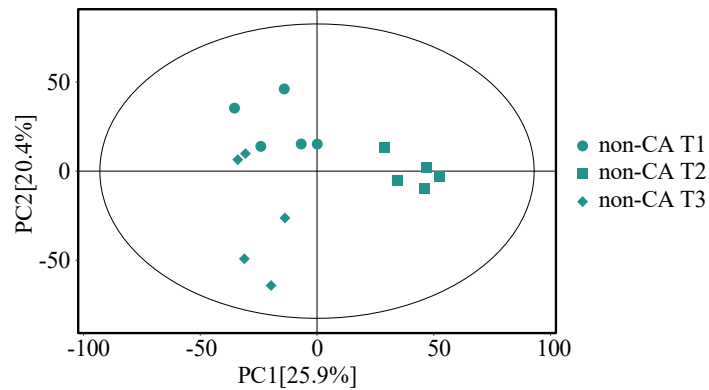

c

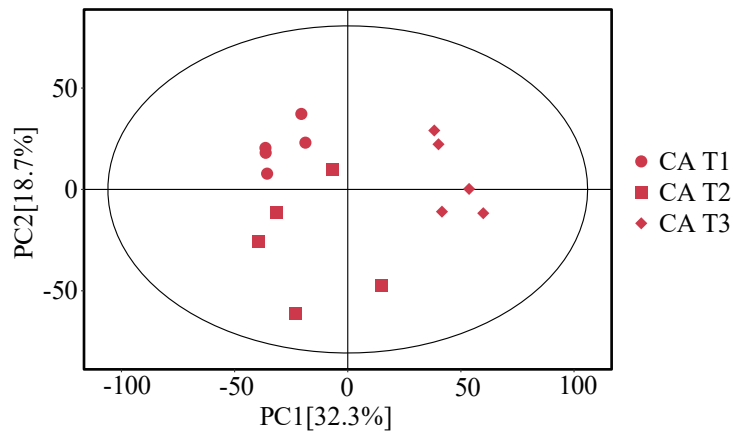

d

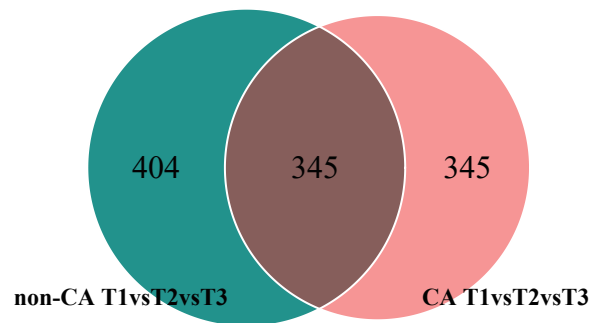

Supplement: Supplementary file 1 [file ijms-25-11189-s001.zip › Figure S5.pdf]
